# Hypersalinity Drives Dramatic Shifts in the Invertebrate Fauna of Estuaries

**DOI:** 10.3390/ani15111629

**Published:** 2025-06-01

**Authors:** Ben J. Roots, Ruth Lim, Stephanie A. Fourie, Essie M. Rodgers, Emily J. Stout, Sorcha Cronin-O’Reilly, James R. Tweedley

**Affiliations:** 1 School of Environmental and Conservation Sciences, College of Environmental and Life Sciences, Murdoch University, 90 South Street, Murdoch, WA 6150, Australiaj.tweedley@murdoch.edu.au (J.R.T.); 2 Centre for Sustainable Aquatic Ecosystems, Harry Butler Institute, Murdoch University, 90 South Street, Murdoch, WA 6150, Australia

**Keywords:** benthic macroinvertebrate, climate change, community composition, diversity, euryhaline, hyperhaline, Mediterranean climate, osmoregulation, salinity gradient, salt lake

## Abstract

In some estuaries, low inflow and/or isolation from the ocean can result in evapoconcentration and hypersalinity (≥40 ppt). This can create osmoregulatory and energetic challenges for the faunal community, leading to reductions in diversity as more species pass their thresholds. As climate change is increasing the magnitude and duration of hypersaline conditions, we used benthic macroinvertebrate data from 12 estuaries across a Mediterranean climatic region (southwestern Australia) to assess the influence of salinity (0–122 ppt) on the invertebrate fauna. Taxa richness and diversity were highest in salinities between 0 and 39 ppt, peaking at salinities closest to seawater, while total density peaked at 40–49 ppt. Beyond 50 ppt, these measures declined significantly. Community composition changed markedly along the salinity gradient. In lower salinities, communities were diverse, comprising polychaetes, malacostracans, hexapods, ostracods, bivalves, and gastropods. However, in salinities ≥50 ppt, many taxa declined, leading to communities dominated by polychaetes (mainly *Capitella* spp.) and hexapods (mostly larval chironomids). At 90 ppt, only polychaetes and hexapods remained, and at ≥110 ppt, only the latter taxon persisted. This faunal shift towards insect dominance in hypersaline conditions mirrors observations in other Mediterranean and arid/semi-arid regions, with the resulting communities resembling saline wetlands or salt lakes. This loss of invertebrates can substantially impact ecosystem functioning and trophic pathways, and the findings of this study provide a basis for predicting how these communities will respond to increasing hypersalinity driven by climate change.

## Simple Summary

Regions with a Mediterranean climate are becoming drier due to climate change and salinities in many estuaries are increasing. Data from 12 estuaries in southwestern Australia were collated and used to assess how the richness, diversity, and community composition of benthic macroinvertebrates changed over a salinity gradient. Taxa richness and various diversity measures were highest in salinities close to seawater and declined sharply in higher salinities. Salinities between 0 and 49 ppt contained a diverse community with species of polychaete worms, bivalves, crustaceans, gastropods, and insects (mainly larvae). However, as salinity increased further, worms, insects, and gastropods dominated until 110 ppt, where only insect larvae remained. These findings are consistent with trends in other hypersaline estuaries and lagoons in Mediterranean and arid/semi-arid climates globally. They highlight a shift towards a simplified community and, in salinities >60 ppt the fauna resembles that of a saline wetland or salt lake, which has significant implications for ecosystem functioning and food webs.

## 1. Introduction

Estuaries are highly productive ecosystems that are of ecological, economic, and societal importance due to the goods and services they provide [1,2]. However, due to anthropogenic influences, these ecosystems are amongst the most degraded and threatened by climate change [3,4,5]. Estuaries support a unique biota due to their nature as a ‘transition zone’, being influenced to different extents by both freshwater and marine environments [6]. The physicochemical conditions in an estuary can vary spatially due to the proximity to freshwater and marine sources, and over a range of temporal scales due to tidal, weather, and climatic patterns [7,8]. Periods of relatively high riverine flow can lower salinity and facilitate the movement of freshwater-associated species into the estuary. Conversely, low riverine flow and/or closure of the sand bar at the mouth of the estuary, combined with evaporation, can increase salinities and favour marine/salt-tolerant species [9]. Salinity is one of the most important variables influencing the survival and distribution of aquatic species, particularly in estuarine environments where salinity can fluctuate substantially over various time scales ranging from hours (e.g., a tidal cycle) to years (e.g., during a drought) [10,11]. It is understood that the consistent osmotic balance of cellular fluid is essential for maintaining homeostasis, including cardiac function, circulation, nerve conduction, and muscle contraction. Hence, a deviation in osmotic pressure that is too large is fatal [12,13]. Estuarine species often have efficient osmoregulatory mechanisms and behavioural adaptations to help them cope with salinity fluctuations [14,15]. However, many species may struggle as human-induced climate change increases the severity, frequency, and duration of these fluctuations.

Human-induced climate change poses a major threat to estuaries globally, albeit the effects differ among regions [16,17]. In Mediterranean climatic regions, e.g., parts of southern Australia, South Africa, the Mediterranean basin and California, rising sea levels, lower annual precipitation and increasing temperatures are reducing freshwater inflow, increasing seawater intrusion, and increasing evaporation in estuaries, resulting in marinisation and increased incidences of hypersalinity (≥40 ppt) [16,18,19]. Such a situation occurs in southwestern Australia, which is a climate change hotspot and where, since the 1970s, rainfall has reduced by 16% and streamflow by 50% [20]. This is especially problematic due to the Mediterranean climate, in which the long, dry summer and autumn months lead to high evaporation [21]. While it is known that Mediterranean climatic regions are drying and that numerous estuaries have become hypersaline for periods, there is limited information on how these conditions might influence invertebrate communities [22].

As hypersalinity typically does not occur suddenly, there would likely be a gradual change in the community structure over time, e.g., contraction in the area of an estuary that a given species can inhabit [23] until that species reaches its salinity tolerance threshold and mass mortalities occur [24]. Due to varying osmoregulatory capacities, energetic demands, and body sizes, salinity tolerance thresholds are not uniform across all species [25]. This disparity can, therefore, lead to distinct community structures under different levels of hypersalinity [26]. Evidence from annual surveys of the Coorong in South Australia for 10 years before and several years after a severe drought showed there was a marked change in the benthic macroinvertebrate fauna [27]. Initially, the system supported a diverse community with numerous polychaete, mollusc, and crustacean species. However, during drought conditions, parts of the system were dominated by chironomid larvae, with 64 ppt identified as the salinity at which the fauna changed. Similarly, it has been noted in a review by Anufriieva and Shadrin [25] that arthropods contributed to 49% of all species in salinities between 35 and 50 ppt, but became increasingly dominant in higher salinities and represented 100% of species recorded in salinities >310 ppt.

A global review highlighted that southwestern Australia was a hotspot for hypersaline estuaries [22], some of which are among the most saline recorded [11,28]. With predictions indicating that winter rainfall will decrease due to climate change [29], it is likely that more estuaries will become hypersaline for periods, and the duration of those conditions will be longer. Although there is some data available on how the structure of the fish community changes with increasing salinity (e.g., [30,31]), such information is lacking for benthic macroinvertebrates. As these species are a crucial component of the fauna, understanding salinity tolerance thresholds of benthic macroinvertebrates would allow for a greater understanding of how hypersalinity impacts and shapes estuarine ecosystems. Pinpointing the salinity thresholds where certain groups of taxa decline can reveal their sensitivity or resilience, and provide insight into food availability for higher-order consumers and/or ecosystem functioning [32] and, in turn, help develop triggers for management action [33]. This study utilised a recently compiled data set of benthic macroinvertebrates across a range of estuaries in southwestern Australia that experience a salinity gradient from oligohaline to markedly hypersaline conditions (0–122 ppt) to determine how the richness, diversity, and composition of the fauna change with salinity. The resultant trends from this study are discussed in the context of findings from hypersaline environments in other Mediterranean and arid/semi-arid regions.

## 2. Materials and Methods

### 2.1. Site Description and Sampling

Southwestern Australia experiences a Mediterranean-type climate, typified by a strong seasonal pattern of cool, wet winters and hot, dry summers [34]. The majority of annual rainfall (>85%) occurs in the cooler months between May and October, with summer rainfall being episodic and driven by deteriorating cyclonic events and thunderstorms [21]. Rainfall and temperature vary across the region and are reflected in the allocation of three Köppen climates, i.e., (i) hot-summer Mediterranean on the upper parts of the western coast, (ii) warm-summer Mediterranean climate on the lower part of the west coast and southern coast until Esperance and (iii) cold semi-arid climate east of Esperance on the southern coast. Rainfall across the region is variable, with the highest mean annual values occurring in the southwest corner (up to ~1400 mm), with a gradient of declining rainfall moving northward and eastward down to ~400 mm [34]. Pan evaporation values are inversely related, ranging from ~1200 mm in the southwest corner to ~2000 mm in the northern and eastern extent of the region. Thus, for most areas of southwestern Australia, evaporation exceeds rainfall [21]. Tides are microtidal and mixed, mainly diurnal, with a maximum daily range of 1.1 m on the lower-west coast and 1.5 m on the southern coast.

**Figure 1:**
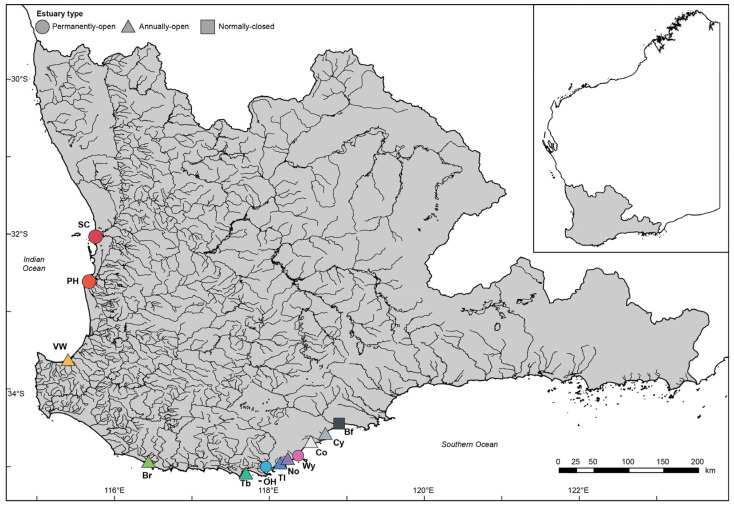
Map of southwestern Australia showing the rivers in the southwest drainage division (grey shading) and the location of the 12 estuaries where samples of benthic macroinvertebrates were collected. SC, Swan-Canning Estuary; PH, Peel-Harvey Estuary; VW, Vasse-Wonnerup Estuary; Br, Broke Inlet; Tb, Torbay Inlet; OH, Oyster Harbour; Tl, Taylor Inlet; No, Normans Inlet; Wy, Waychinicup Estuary; Co, Cordinup River; Cy, Cheyne Inlet; Bf, Beaufort Inlet. Insert shows the location of the drainage division within Western Australia. Taken from Lim et al. [36].

There are ~50 estuaries in southwestern Australia [21,35], which can be grouped into three broad categories based on the extent of their connectivity to the ocean: (i) permanently open, i.e., the sand bars at their mouths never close; (ii) annually-open, i.e., sand bars close during periods of low inflow; however, they open to the ocean for part of each year; and (iii) normally closed, i.e., sand bars may be closed for several years at a time. This study utilised a data set collated by Lim et al. [36], from 12 estuaries distributed along the southwestern Australian coastline that cover all three categories (Figure 1). Each estuary has unique physical characteristics, including catchment and estuary size, volume of freshwater inflow, sediment characteristics, and submerged aquatic vegetation (Table S1). Due to these differences, they experience marked variations in salinity ranging from ~0 to 122 ppt (Figure S1).

Full details of the sampling protocols are provided in Lim et al. [36], the relevant source publications [4,37,38,39,40,41,42,43,44], and are summarised briefly in Table S2. In brief, sampling was conducted along the longitudinal axis in all estuaries to incorporate the full range of salinities experienced during sampling. Moreover, the sites (12–60 per estuary) encompassed a range of sediment types and areas with and without submerged aquatic vegetation (e.g., seagrass and charophytes). Sampling was conducted between two and 15 times in each estuary to account for temporal variation. This extensive sampling yielded 1891 samples, with between 48 and 480 samples from each estuary. To ensure consistency in identification, the reference collections from the various studies were examined, and the taxonomy of all 259 taxa was checked and updated where necessary using the World Register of Marine Species [45]. The abundance of each macroinvertebrate taxon in each replicate sample was converted into a density (100 cm^−2^).

### 2.2. Data Analysis

All analyses were conducted using the PRIMER v7 multivariate statistics software package [46], except where stated. The data matrix of the abundance of each macroinvertebrate taxon in each sample was used by DIVERSE to calculate the number of taxa (taxa richness), Shannon diversity, Simpson’s index (1 − λ′), total density (100 cm^−2^), and quantitative taxonomic distinctness (Δ*). The mean and 95% confidence intervals were calculated and plotted for each salinity category (10 ppt intervals; i.e., 0 = 0.0 to 9.9, 10 = 10 to 19.9 ppt, and so on). Note that due to differences in the number of samples in each category, no statistical analyses were conducted. The data matrix was then averaged for each salinity category and subjected to TAXDTEST to determine the ‘expected’ value and 95% probability limits for qualitative taxonomic distinctness (Δ^+^) in random subsamples of different numbers of taxa drawn from the full suite of 259 taxa found in southwestern Australian estuaries. These data were used to construct a funnel plot onto which the measured values of Δ^+^ were superimposed, both to compare values and to test for any significant departures from expectation, i.e., lie outside the 95% probability limits [47]. The values for Δ^+^ and the number of taxa were subjected to a one-tailed correlation in SPSS v29 to determine whether there was a significant relationship. As Δ^+^ is independent of the number of species [48], a negative relationship would indicate that species are not lost across all taxa equally, but differentially from particular higher taxonomic groups [37]. Spearman’s correlation was used as the values for Δ^+^ were not normally distributed (Kolmogorov–Smirnov *p* ≤ 0.001).

The average abundance of each macroinvertebrate taxon in each salinity category was aggregated to the phylum and class levels, standardised to calculate percentage composition and used to construct stacked bar graphs. Note for clarity, only phyla and classes that represented ≥1% in any of the salinity categories were included. These groups represented between 99.5 and 100% of all individuals in a category. The data in each matrix were then square-root transformed to down-weight the contributions of dominant taxa and produce a Bray–Curtis resemblance matrix. In turn, the matrices were used to create a non-metric Multidimensional Scaling (nMDS) ordination plot and subjected to hierarchical agglomerative cluster analysis to produce a dendrogram [46]. Superimposed on the nMDS ordinations was a trajectory denoting the linear change in salinity categories. This seriation was quantitatively examined using a RELATE test, with the magnitude of any significant relationship (*p* < 0.05) calculated using the Rho statistic (ρ), which ranges from ~0 (little correlation) to 1 (perfect correlation) [46]. An nMDS (with associated RELATE test) and a cluster dendrogram were produced using the square-root transformed species composition data.

Coherent species curves [49] were used to investigate how the percentage composition of individual taxa changed across the salinity categories and groups those with statistically similar patterns (*p* < 0.05; i.e., coherent groups of taxa) using cluster analysis. As taxa only found sporadically and in relatively low abundances will only add random noise to the similarities, only the 50 most ‘important’ taxa, i.e., those that made the largest contributions to at least one of the salinity categories, were included. [50]. The percentage contribution of each of these taxa in each of the salinity categories was displayed in a shade plot [51], onto which the cluster dendrogram was overlaid.

## 3. Results

### 3.1. Univariate Diversity Measures

**Figure 2:**
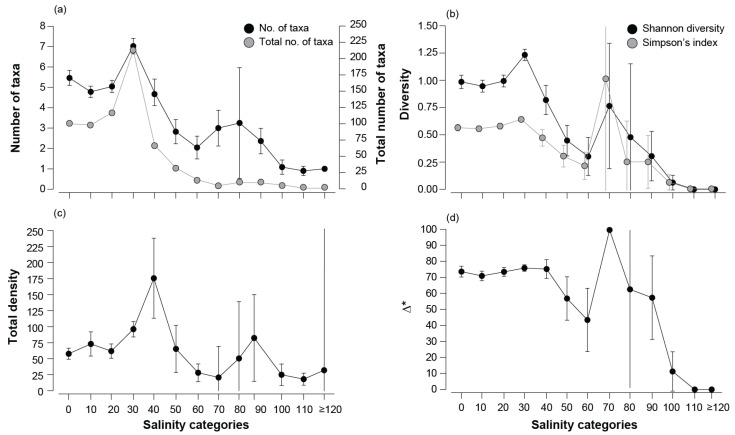
(**a**) Total number of taxa and mean values (± 95% confidence limits) of the number of taxa, and mean values (± 95% confidence limits) of (**b**) Shannon diversity and Simpson’s index, (**c**) total density (individuals 100 cm^−2^), and (**d**) average quantitative taxonomic distinctness (Δ*) of the benthic macroinvertebrate community in each salinity category (10 ppt intervals; i.e., 0 = 0.0 to 9.9, 10 = 10 to 19.9 ppt, and so on).

A total of 259 benthic macroinvertebrate taxa were recorded across the 12 estuaries. While these taxa represented 11 phyla, 94% were arthropods (118 taxa, including 58 malacostracans and 47 hexapods), annelids (83 taxa, including 78 polychaetes), and molluscs (43 taxa, including 24 gastropods and 16 bivalves). The total number of taxa was ~100 in the 0 ppt (i.e., 0–9.9 ppt) and 10 ppt categories, increased to 117 (20 ppt), and substantially to 212 in the 30 ppt category (Figure 2a). Values declined substantially to 67, 32, and 14 taxa in the 40, 50, and 60 ppt categories, respectively, before remaining relatively consistent at between 5 and 10 taxa in the 70 to 100 ppt categories, with only a single species recorded in 110 and ≥120 ppt.

The mean values for the number of taxa, Shannon diversity, and Simpson’s index displayed similar trends, with moderately high values at the 0, 10, and 20 ppt categories, reaching a peak at 30 ppt (Figure 2a,b). Values of all three measures declined sequentially to 60 ppt before rising to a second peak at 70 ppt and then declining again. Similarly, their lowest values occurred in salinity categories of 100, 110, and ≥120 ppt. Mean total density displayed a different pattern with values of between ~55 and 100 indiv. 100 cm^−2^ in salinity categories of 0 to 50 ppt, with a peak of ~176 indiv. 100 cm^−2^ in 40 ppt (Figure 2c). Mean densities in categories >60 ppt were 18–50 indiv. 100 cm^−2^, except for a second peak of 50 and 80 indiv. 100 cm^−2^ in the 80 and 90 ppt categories, respectively. The trends in mean quantitative taxonomic distinctness (∆*) differed from the other diversity measures, being similar in all categories up to 40 ppt (i.e., 70–75). They then declined at 60 ppt before increasing to a peak at 70 ppt (100) and then declined to ~60 at 80 and 90 ppt and then sharply to 11 at 100 ppt and 0 in both 110 and ≥120 ppt (Figure 2d).

**Figure 3:**
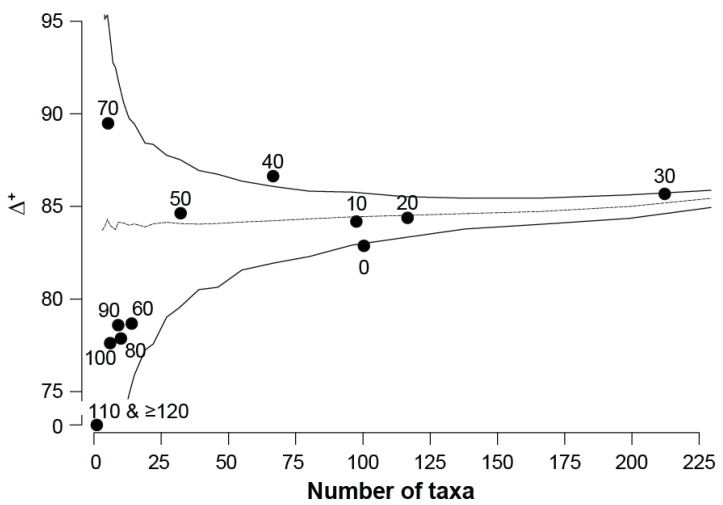
Funnel plot depicting the qualitative taxonomic distinctness (Δ^+^) of each salinity category (10 ppt intervals; i.e., 0 = 0.0 to 9.9, 10 = 10 to 19.9 ppt, and so on) plotted against the number of taxa found in that category. The dashed line denotes the ‘expected’ value derived from data for random subsamples of taxa found in the estuaries, and the black lines the upper and lower 95% probability limits. Note that the ∆^+^ values for the 110 and ≥120 ppt salinity categories were 0.

Across the salinity gradient, the pattern of qualitative taxonomic distinctness (∆^+^) values was similar to that of quantitative taxonomic distinctness. Values ranged between 83 and 87 in the categories between 0 and 50 ppt, before declining to ~78 in categories between 60 and 100 ppt (except for 70 ppt) and then being 0 at 110 and ≥120 ppt (Figure 3). On the funnel plot, all values of ∆^+^ fall within the 95% probability limits, except for the 0, 40, 110, and ≥120 ppt categories. Thus, the taxa recorded in most salinity categories have a level of taxonomic distinctness fully representative of the regional species pool, with that of 40 ppt being significantly greater and those of 0, 100, and ≥120 ppt significantly lower. Among those within the funnel, the ∆^+^ values for 30, 50, and particularly the 70 ppt category are above the mean, while those for the 60, 80, 90, and 100 ppt are considerably below the mean (Figure 3). There was a significant positive correlation between ∆^+^ and the number of taxa (ρ = 0.581; *p* = 0.019).

### 3.2. Community Composition

**Figure 4:**
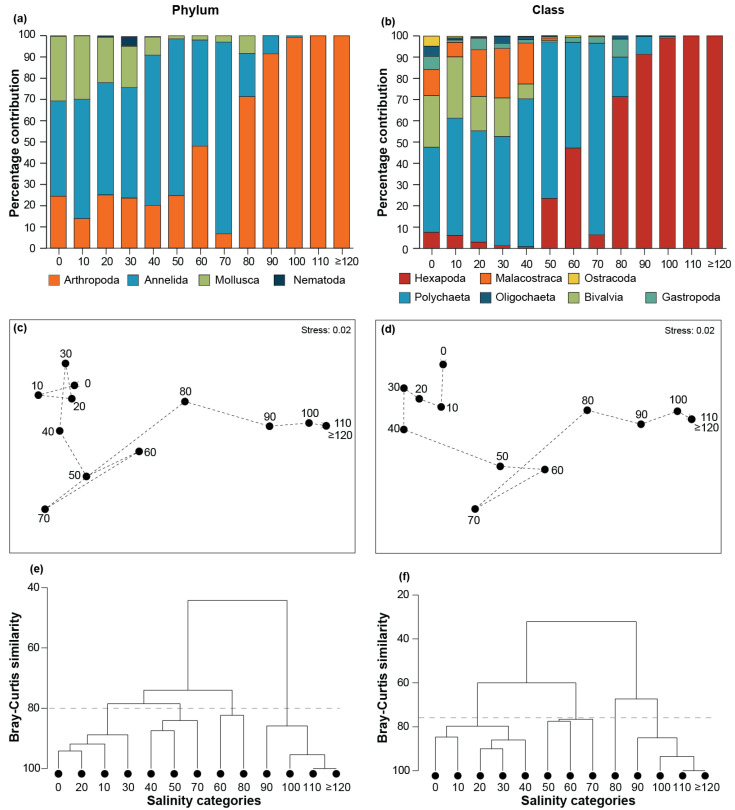
Percentage contribution of individual benthic macroinvertebrates belonging to various (**a**) phyla and (**b**) classes in each salinity category (10 ppt intervals; i.e., 0 = 0.0 to 9.9, 10 = 10 to 19.9 ppt, and so on). (**c**,**d**) nMDS ordination plots, and (**e**,**f**) cluster dendrograms of the percentage composition of the benthic macroinvertebrate community at the phylum and class levels, respectively, in each salinity category. Dashed lines in (**c**) and (**d**) denote successive salinity categories, and in (**e**) and (**f**) Bray–Curtis similarities of 80 and 76, respectively.

Annelida was the dominant phylum by the relative percentage contribution of individuals in the 0 to 70 ppt salinity categories, contributing ~45% in 0 ppt, >50% in 10, 20, and 30 ppt, and >70% in 40 and 50 ppt categories (Figure 4a). The contribution of annelids declined to 50% in 60 ppt before increasing to its maximum contribution of ~90% in 70 ppt, followed by a decline to 20%, 9%, and <1% in the 80, 90, and 100 ppt categories, respectively. Molluscs made the second highest contribution (~30%) in the 0 and 10 ppt categories; however, their contribution declined in higher salinities (i.e., 1 to 21%) with arthropods being more numerous. Arthropods surpassed annelids as the dominant contributors in all categories ≥80 ppt, where they comprised between 71 and 100% of all individuals. Molluscs were not recorded in categories ≥90 ppt, and neither were annelids in those ≥110 ppt (Figure 4a). Of the four phyla, nematodes made the lowest contribution, with a peak in contribution of ~5% in the 30 ppt category, and were not recorded in categories ≥50 ppt. Echinoderms were only recorded in the 30 ppt category and made a very minor contribution (0.03%, not illustrated in Figure 4a).

When assessing the relative percentage contribution by class, polychaetes were dominant in all salinity categories ≤70 ppt. This class contributed 40% of individuals in 0 ppt, ~50% in the 10, 20 and 30 ppt categories, 70% and 74% in the 40 and 50 ppt categories, respectively, and peaked at 90% contribution at 70 ppt, after which it began to decline until it was not recorded in salinities ≥110 ppt (Figure 4b). Bivalves made the second highest contribution of 24 and 29% in the 0 and 10 ppt categories, respectively. However, in higher salinities, they were surpassed by the malacostracans, who contributed ~20% in the 20, 30, and 40 ppt categories. Bivalves were not recorded in categories ≥60 ppt, while malacostracans were scarcely found above this salinity and were completely absent in the 110 and 120 ppt categories. Hexapods made the second highest contribution in categories ≥50 ppt and became the dominant class in the 80 ppt category and above, being the only class recorded in the 110 and ≥120 ppt categories. Gastropods were recorded in all categories between 0 and 80 ppt, making their largest contribution (8%) in the latter category. Minor contributions were also made by oligochaetes in the 0, 10, 30, 40, and 80 ppt categories (1–5%) and by ostracods (5%) in 0 ppt (Figure 4b).

There was a significant serial change in composition at the phylum level (ρ = 0.771; *p* = 0.001), and there is a transition in the arrangement of salinity categories from left to right across the nMDS plot (Figure 4c). At the phylum level, four groups were evident at a Bray–Curtis similarity of 80, i.e., (i) 0 to 30 ppt, (ii) 40, 50, and 70 ppt, (iii) 60 and 80 ppt, and (iv) 90 to ≥120 ppt (Figure 4e). Categories in the first group were tightly clustered (Bray–Curtis similarity = 85–94) and all were dominated by annelids, with relatively equal contributions from arthropods and molluscs. The 40, 50, and 70 ppt group was broader and was distinguished from the 0 to 30 ppt group by the increased contributions of annelids and a corresponding reduction in molluscs (Figure 4a). The group comprising the 60 and 80 ppt categories was more dispersed and located in the centre of the nMDS plot. Arthropods dominated these categories, but substantial numbers of annelids were present. The remaining group comprised a very tight cluster containing the 100, 110, and ≥120 ppt categories (Bray–Curtis similarity = 95–100) and the 90 ppt category that was slightly less similar (84–89). This was due to the three highest salinity categories comprising exclusively (110 and ≥120 ppt) and almost exclusively (100 ppt) arthropods, whereas annelids made a small contribution (10%) to the fauna in the 90 ppt category.

The serial change in composition was slightly stronger at the class level (ρ = 0.831; *p* = 0.001), due to the position of the 60 ppt category with 50 and 70 ppt rather than 80 ppt (Figure 4e,f). At a Bray–Curtis similarity of 76, four groups were distinguished, i.e., (i) 0 to 40 ppt, (ii) 50 to 70 ppt, (iii) 80 ppt, and (iv) 90 to ≥120 ppt. Polychaetes dominated the fauna in the first group of categories, albeit with several other classes, e.g., bivalves, malacostraceans, and gastropods, that made substantial contributions. The contribution of annelids increased in the 50, 60, and 70 ppt categories, with hexapods comprising most of the remaining individuals. These two classes also dominated the fauna in the 80 ppt category, only with hexapods representing the majority. Finally, hexapods comprised almost all or all of the individuals in the 90 to ≥120 ppt categories.

**Figure 5:**
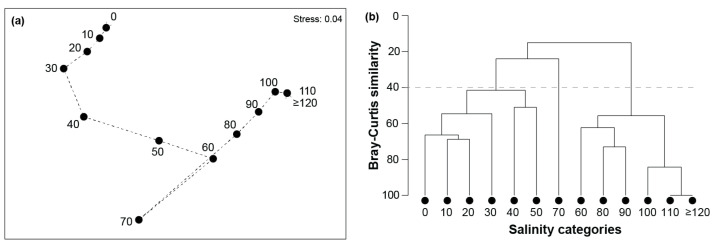
(**a**) nMDS and (**b**) cluster dendrogram of the percentage composition of species to the benthic macroinvertebrate community in each salinity category (10 ppt intervals; i.e., 0 = 0.0 to 9.9, 10 = 10 to 19.9 ppt, and so on). Dashed lines in (**a**) denote successive salinity categories, and in (**b**) a Bray–Curtis similarity of 40.

**Figure 6:**
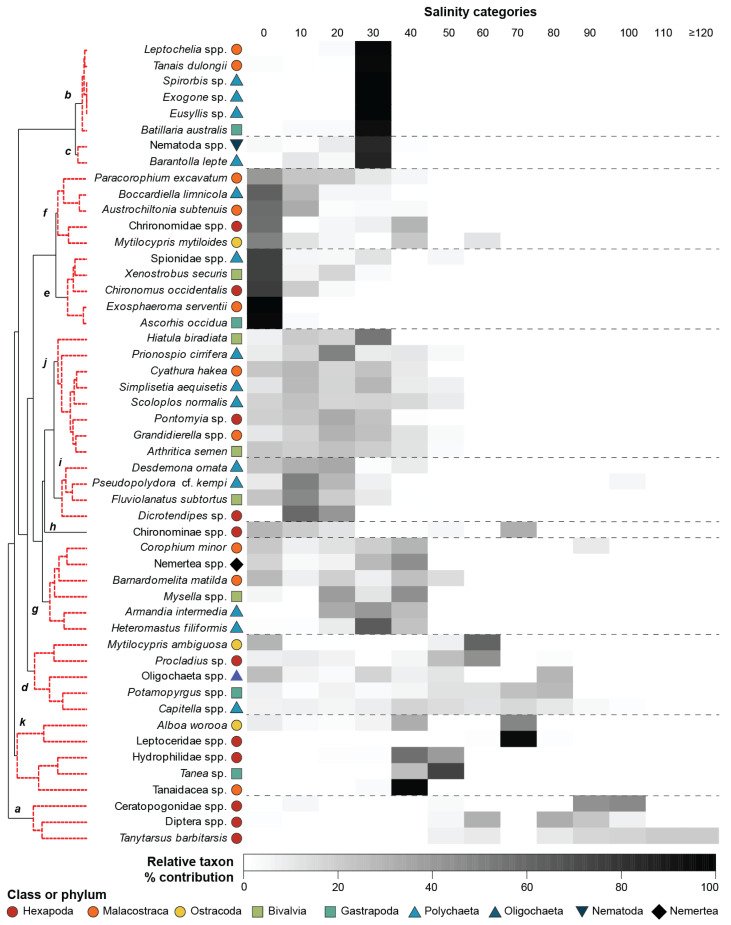
Shade plot of the percentage contribution of each of the 50 most important benthic macroinvertebrate taxa among salinity categories (10 ppt intervals; i.e., 0 = 0.0 to 9.9, 10 = 10 to 19.9 ppt, and so on). Taxa joined by dashed red lines on the dendrogram represent a coherent group of taxa (bold italics), i.e., have a statistically similar pattern of contribution across the salinity categories.

The extent of seriation at the species (taxa) level was the same as that at the class level (ρ = 0.831; *p* = 0.001). This was clearly illustrated on the nMDS plot, where all the categories except 70 ppt were arranged in sequential order from left to right (Figure 5a). Although there was evidence of two clear groups of salinity categories on the nMDS plot and cluster dendrogram, i.e., (i) 0 to 50 ppt and (ii) 60, 70 and ≥80 ppt, with 70 ppt as an outlier, examination of the Bray–Curtis similarities and the coherent species curves suggests a more nuanced situation. There was, as with the other taxonomic levels, relatively high similarity among the 0, 10, and 20 ppt categories (Bray–Curtis similarity = 65–69); however, there was a more pronounced reduction in the similarity of the 30 ppt category to those (i.e., 51–61). A broad suite of taxa in coherent species groups *d*, *e*, *f*, *g*, *h*, *j*, and *i* were found in all salinity categories from 0 to 30 ppt (Figure 6), indicating they are relatively euryhaline. However, the contributions of taxa in groups *e* (e.g., the chironomid *Chironomus occidentalis*), *f* (e.g., the ostracod *Mytilocypris mytiliodes* and polychaete *Boccardiella limnicola*), and *i* (e.g., polychaete *Desdemona ornata*) declined as salinities reached 30 ppt (Tables S3 and S4). In contrast, stenohaline marine taxa in coherent group *b* (e.g., the gastropod *Batillaria australis*) and more widely occurring taxa in group *c* (i.e., nematodes and the polychaete *Barantolla lepte*) were more abundant in marine-like salinities. The salinity categories 40 and 50 ppt were less similar to those between 0 and 30 ppt (i.e., on average 51 and 32, respectively). While key taxa in these two hypersaline categories were still present in substantial abundances, e.g., coherent groups *g* (e.g., the amphipod *Barnardomelita matilda*), *j* (i.e., the polychaete *Prionospio cirrifera*), and *k* (e.g., the water beetle Hydrophilidae spp. and ostracod *Alboa worooa*), taxa in coherent groups *b*, *c* and *i* declined markedly.

Within the second broad cluster of salinity categories, there was also evidence of a stepwise change in faunal composition from a group of 60, 80, and 90 ppt to 100 ppt and finally 110 and ≥120 ppt (Figure 5b). This was due to a successive loss of taxa as salinity increased. In the 60 ppt category, the community was mostly dominated by the chironomid *Tanytarsus barbitarsis* and Diptera spp. (both in coherent group *a*), with smaller abundances of the gastropod *Potamopyrgus* spp., another chironomid *Procladius* sp., together with *M. ambiguosa* and *Capitella* spp. (coherent group *d*). In salinity categories 80 and 90 ppt, only *T. barbitarsis*, Diptera spp., and another dipteran, Ceratopogonidae spp., were relatively abundant, albeit *Capitella* spp. was also present (Figure 6). *Tanytarsus barbitarsis* was the only species recorded in salinities ≥110 ppt.

## 4. Discussion

With human-induced climate change continuing to decrease rainfall in Mediterranean regions of the world and anthropogenic alterations to flow regime, the frequency and magnitude of hypersalinity (≥40 ppt) in many estuaries will increase. Given their crucial role, considerations of the impacts of hypersalinity on benthic invertebrates must be taken to ensure adequate ecosystem functioning and maintenance of trophic processes. Understanding the shifts in community composition from freshwater to extremely hypersaline conditions based on species salinity tolerances is essential for interpreting such impacts. The richness, diversity, and community composition of benthic macroinvertebrates within 12 estuaries of southwestern Australia changed markedly across the salinity gradient, with distinct communities occurring in certain salinity ranges. Taxa richness and diversity were highest in the 0 to 30 ppt categories (i.e., 0–39 ppt) and reached their maximum in salinities closest to seawater (i.e., 30 ppt category); meanwhile, total density was highest in the 40 ppt category. In categories ≥50 ppt, these measures all declined. When assessed at a phylum and class level, community composition was similar in 0 to 40 ppt salinity categories and consisted of a diverse range of annelid (polychaetes), arthropod (malacostracans), and molluscan (bivalves) taxa. This was followed by a shift to a more depauperate composition in the 50 to 70 ppt categories, generally made of annelids (mostly polychaetes), some gastropods, and arthropods (hexapods and malacostracans). In salinities ≥80 ppt, the faunal community comprised mainly hexapods.

### 4.1. Impacts of Hypersalinity on Diversity

**Figure 7:**
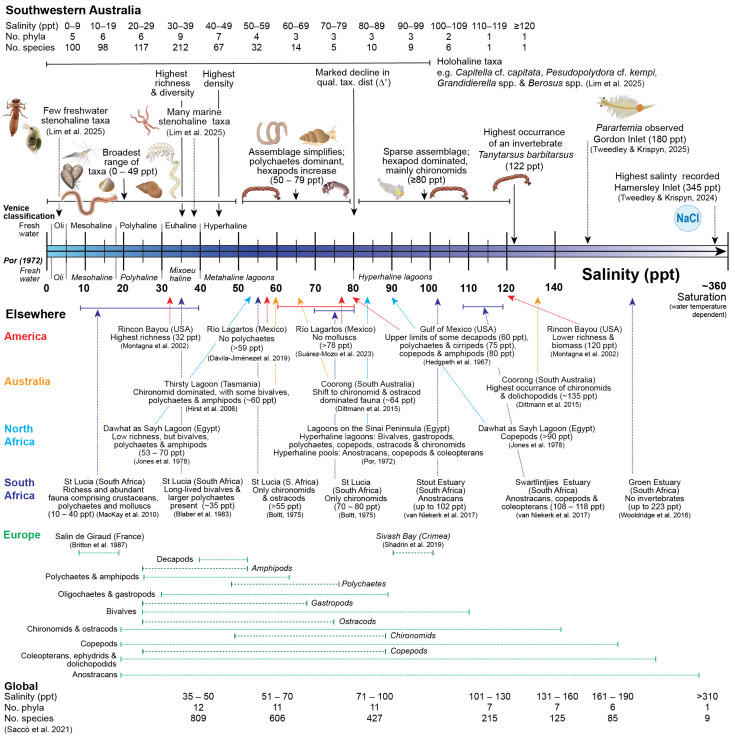
Conceptual diagram summarising how aspects of the benthic macroinvertebrate fauna of estuaries in southwestern Australia (top) and in other Mediterranean and arid/semi-arid climates (bottom) change along the salinity gradient with a focus on hypersalinity [9,27,28,36,54,55,58,60,69,70,71,76,77,79,80,81,82,83].

Shifts in the richness, density, and community composition of benthic macroinvertebrates have been recorded with increasing salinity in this study and are similar to those in estuaries and lagoons in Mediterranean and arid/semi-arid climates (Figure 7). In keeping with the Rename diagram, there was a sequential increase in the number of phyla and species across the categories from oligohaline (0–9 ppt) to euhaline (30–39 ppt) waters [52], reflecting an increase in the number of stenohaline marine species [36]. However, the number of phyla and species declines rapidly and sequentially (except 70 ppt) in hypersaline salinities, with only a single species, i.e., larvae of the chironomid *Tanytarsus barbitarsus* in ≥120 ppt. This mirrors the sequential pattern of richness in hypersaline ecosystems globally (Figure 7; [53]) and of particular estuaries, e.g., Rincon Bayo (USA) and St Lucia (South Africa) [54,55], caused by the loss of species as they reach the upper limit of their salinity tolerance. For example, in St Lucia Boltt [9] recorded 21 species in salinities of 45–58 ppt, 9 in 55–60 ppt, and only one (a chironomid) in 70–80 ppt. In hypersaline water bodies across the Crimean Peninsula, chironomids dominated, comprising >70% of the total number of taxa, and reaching peaks in density at 150–170 ppt [56]. Even within the Chironomidae family, species richness declines as salinity rises [57]. In some cases, extreme levels of salinity can result in no invertebrates being recorded, as occurred in the Groen Estuary (South Africa), where salinities reached 223 ppt [58]. Part of the reduction in richness seen in the current study could be the result of bar closure in many southwestern Australian estuaries, as closure prevents the recruitment of species from the marine environment, hence lowering the number of species present [59]. However, many marine species are stenohaline [52] and hypersaline conditions lead to a reduction in species richness in systems elsewhere that are permanently open to the ocean, e.g., the Laguna Madre (USA) and Coorong (Australia) [27,60]. Despite the reduction in richness in salinity categories ≥40 ppt in the current study, the two taxonomic distinctness measures (Δ* and Δ^+^) did not typically decline until 60 ppt. This is due, in part, to several species in the major taxonomic groups occurring in high salinities, e.g., the bivalve *Arthritica semen* (54 ppt), the gastropod *Potamopyrgus* spp. (97 ppt), the amphipod *Grandidierella* spp. (which contained *G. japonica* and *G. propodentata*; 101 ppt) and the polychaete *Capitella* spp. (including *Capitella capitata;* 103 ppt) [36]. The positive relationship between qualitative taxonomic distinctness and the number of taxa does indicate, however, that despite these exceptions, at higher salinities, taxa belonging to particular groups are more sensitive to hypersalinity.

Ecological theory suggests that a greater taxonomic biodiversity will increase the number of expressed traits, resulting in greater functional diversity (i.e., functional richness, functional evenness, and functional redundancy) and therefore exhibit larger effects on ecosystem functioning [61]. In the current study, there was an essentially sequential reduction in richness at the phylum and species levels as the magnitude of hypersalinity increased, which, following other studies, could indicate that aspects of functional diversity would decrease too [62,63]. Moreover, Lam-Gordillo et al. [61] found that functional redundancy in the Coorong was lower than in nearby coastal ecosystems and suggested this made it less resistant to further loss of benthic taxa and more likely to lose ecosystem functioning.

Mean total density displayed a different trend to that exhibited by the other diversity measures, with a major peak at 40 ppt (rather than 30 ppt), followed by a decline to 70 ppt, before rising to a secondary, although lesser, peak at 90 ppt. The increased density after the declines of other diversity measures could be the result of decreased competition between taxa, allowing for an increase in the abundance of the remaining opportunistic species [64], such as *Capitella* spp., which typified the fauna in the 40 ppt category. Moreover, the estuaries in southwestern Australia become hypersaline through evaporation during periods of bar closure, which not only concentrates salt but also nutrients in the water column, often leading to eutrophication and algal blooms [65,66,67]. These conditions allow the remaining salt-tolerant and opportunistic taxa to exploit the resources in a less competitive environment with limited predation and increase their abundance [64]. This accounts for the peaks in density; however, a threshold is still reached when salinities increase too far, even for the more salt-tolerant species, reducing densities again.

### 4.2. Impacts of Hypersalinity on Community Composition

In this study, and in other estuaries and lagoons that become hypersaline, euhaline salinities were found to support the most diverse fauna, comprising annelids, malacostracans, and molluscs [27,55]. By comparing the trends in the current study from southwestern Australia to those in other parts of the world, a general trend in faunal shift emerges (Figure 7). Echinoderms are among the first species to decline (see also [36]), as most are not able to osmoregulate [68]. In some parts of the world, e.g., Río Lagartos (Mexico) and Salin de Giraud (France), polychaetes were the next major taxa to decline, not being recorded in salinities >59 and >63 ppt, respectively [69,70]. The contribution of polychaetes to the invertebrate community in southwestern Australia did not decline until higher salinities, mainly due to large abundances of *Capitella* spp. Similarly, individuals in this species complex have been recorded making substantial contributions to the fauna of the Coorong in salinities ~100 ppt [27]. No molluscs were recorded in the Río Lagartos in salinities >78 ppt [71], and the last substantial contribution from a mollusc in the current study was the *Potamopyrgus* spp. (97 ppt), which is known to inhabit saline wetlands [72]. Another gastropod, *Coxiella striata*, was only recorded in low abundances in salinities up to 70 ppt, but is halophilic and common in Australian salt lakes [73]. The lower contribution of bivalves in hypersaline salinities is due to a decline in the abundance of *A. semen.* This is a small-bodied species that can reach densities of up to 45,000 m^−2^ in the Peel-Harvey Estuary and was present in salinities consistently up to 75 ppt (and in one instance 125 ppt) in the Coorong [74,75]. Salinity tolerances vary greatly among species of malacostracans and hexapods. Most of the abundant malacostracans in the current study were amphipods, which are more tolerant of salinity than decapods (Figure 7, [60,70]). Amphipods declined in abundance in salinities ≥50 ppt, despite low numbers of some taxa, e.g., *Grandidierella* spp. [36] being present in more saline conditions. In the case of *Grandidierella*, many species in this genus are found in hypersaline waters in Europe, Asia, Africa, and Australia [36].

In this study and the literature, there was a clear shift in composition from a diverse fauna comprising substantial contributions of polychaetes, molluscs, and a broad range of malacostracans, to one dominated by arthropod taxa such as chironomids, ostracods, coleopterans, and, in some cases, anostracans (Figure 7). For example, sampling in the Sout and Swartlintjies estuaries (South Africa) in salinities up to 102 ppt and 108–118 ppt, respectively, yielded mainly *Artemia*, copepods, and hydrophilid beetles [76], and in St Lucia in 70–80 ppt larvae of only a single chironomid species were recorded [9]. Although not found in this study, species of *Parartemia* have been observed in Gordon Inlet (Australia), at 180 ppt [77], and were found in the diet of the sparid Black Bream (*Acanthopagrus butcheri*) in Culham Inlet (Australia), when salinities were >85 ppt [78]. This suggests that certain estuaries in southwestern Australia provide, at times, suitable conditions for anostracans. In the current study, the faunal shift towards an arthropod-dominated community occurred in different salinity categories depending on the taxonomic resolution, which, at the species level, was 60 ppt. This is similar to numerous studies elsewhere. For example, in St Lucia, chironomids and ostracods were the only invertebrates recorded in salinities >55 ppt, and in Thirsty Lagoon (Australia), chironomids dominated the fauna in areas of ~60 ppt [9,79]. Quantitative analysis of a ten-year data set for the Coorong (Australia), including during a major drought and a flood event, revealed a distinct change in the benthic community at 64 ppt, with higher salinities being dominated byinsect larvae (predominantly chironomids) and ostracods [27]. Stemming from those findings and that of Wooldridge et al. [58], a salinity threshold of 60–65 ppt for triggering a distinct shift in invertebrate communities to one where insect larvae were the dominant taxa was suggested by Cronin-O’Reilly et al. [4] for Beaufort Inlet (Australia).

In all these studies, the invertebrate community after the faunal shift appears to resemble less of an estuarine community (euhaline conditions) and more of a saline wetland and/or salt lake [84,85,86]. The latter ecosystems are typified by hardy, halophilic species such as ostracods, copepods, anostracans, and dipteran and coleopteran larvae [73]. These species are capable of surviving much higher salinities and typically have life cycles that allow for them to escape the most extreme salinities/drying periods (i.e., cysts or non-aquatic and flying life stages) and return when levels are more tolerable [87]. In the case of insect larvae, the recolonisation of an estuary after a hypersaline episode could also be aided by the inadvertent dispersal of non-flying larvae by birds [88]. With invertebrates being integral to ecosystem functioning and a key link in trophic pathways [89,90], the loss or reduced abundance of a species can lead to complications for the provision of ecosystem goods and services and impact higher-order species [32].

The marked shift in faunal composition will also likely impact the suite of traits that the occurring species possess. For example, Blaber et al. [83] noted that long-lived bivalves and large polychaetes were present in St Lucia after a period of stable euhaline salinities (~35) that were absent during a previous extended period of hypersalinity. Invertebrates are also ecosystem engineers, with species of shellfish (e.g., ostreids and mytilids) and polychaetes (e.g., sabellariids, terebellids, and serpulids) building biogenic reefs. The reefs provide a substrate for sessile biota, shelter and foraging habitat for epifauna, and ecosystem services such as water filtration and fisheries production [91,92]. However, few mytilids were recorded in southwestern Australian estuaries in hypersaline conditions, and species such as *Xenostrobus securis* are some of the molluscs that were present in mesohaline and euhaline salinities but not hypersaline ones. Thus, prolonged periods of hypersalinity that individuals could not avoid by valve closing and/or occurring during spawning could result in the degradation or loss of shellfish habitat. Similarly, reefs of the polychaete *Ficopomatus enigmaticus* are present in the Vasse-Wonnerup and Beaufort estuaries in southwestern Australia and in the Coorong [93]. However, in the latter system, no reefs are present in the South Lagoon, which has been attributed to extreme hypersalinity [93].

Not only does hypersalinity reduce functioning, but it can also lead to alterations in dietary composition and a simplification in the trophic pathways [64]. This alteration has been observed in the diet of the opportunistic *A. butcheri* in the normally closed Stokes, Hamersley, and Culham Inlets in southwestern Australia that exhibited different levels of hypersalinity [78]. In the Stokes Inlet (the least hypersaline, i.e., a maximum of ~50 ppt), *A. butcheri* possessed a diet consisting predominantly of macrophytes, polychaetes, and crustaceans. However, in Culham Inlet, which exceeded >200 ppt (although *A. butheri* can only survive in salinities ≤100 ppt [30]), the diet of this sparid comprised a greater contribution of insects (mostly chironomids) and euryhaline teleosts, along with small volumes of *Capitella capitata* and *Parartemia*. The narrower dietary breadth and switch in prey to insects and teleosts could be due to a reduction in most polychaetes and crustaceans in salinities >50 ppt, as occurred in the current study.

### 4.3. Future Directions

Estuaries and lagoons in Mediterranean climatic regions are becoming increasingly saline due to reduced precipitation from climate change and anthropogenic impacts such as water diversion and secondary salinisation [34]. However, as salinity is not the only factor influencing the fauna [25], there is a need to investigate, through a combination of field observations and controlled laboratory experiments, the individual impact of salinity and the potential synergistic impacts of other climate change and anthropogenic stressors (i.e., high temperature, low dissolved oxygen, pH, and high nutrient levels). While this study compiled 1891 samples from 12 estuaries in southwestern Australia to create a representation of how the richness, diversity, and composition of the benthic macroinvertebrate community changed across a large salinity gradient, it does not encapsulate all specific salinity levels. There were fewer samples collected in salinities >50 ppt (and particularly the 70 ppt category). More samples in these categories would allow the analyses conducted here to occur at a finer resolution (e.g., 5 ppt) and also allow the potential influence of covariates to be incorporated. Further research and monitoring should focus on the estuaries currently threatened by hypersalinity. Given the longitudinal shift in rainfall across southwestern Australia, the knowledge gained from understanding the effects on these systems may be applicable to others as rainfall decreases in the future and more areas become hypersaline.

## 5. Conclusions

Salinity is an important factor shaping the benthic invertebrate community in southwestern Australian estuaries. Taxa richness and diversity measures are highest in salinities close to seawater, after which they decrease rapidly. Density displays a slightly different trend, peaking later than diversity, potentially highlighting the exploitation of resources by salt-tolerant opportunistic species under reduced competition. In oligohaline to euhaline waters, the invertebrate community comprised a diverse range of polychaetes, malacostracans, hexapods, bivalves, gastropods, and ostracods. However, when salinities exceeded 50 ppt, many taxa declined, and the community simplified, becoming dominated by polychaetes (predominantly *Capitella* spp.) and hexapods (mostly larval chironomids) with small abundances of the salt-tolerant gastropods. By 90 ppt, only polychaetes and hexapods remained, and in ≥110 ppt, only hexapods persisted. Similar results have been observed in other estuaries and lagoons in Mediterranean and arid/semi-arid climates globally, with the fauna in many systems shifting to a community dominated by hexapods, ostracods, copepods, and anostracans. These faunal communities resemble less of an estuary and more of a saline wetland and/or salt lake, typified by halophilic taxa. The loss of invertebrates can have a myriad of impacts on the environment, both due to their importance for ecosystem functioning and to trophic pathways. The data presented in this study can be used to make predictions as to how benthic invertebrate communities in estuaries in Mediterranean and arid/semi-arid climates may change with increasing hypersalinity.

## Data Availability

The data presented in this study are available on request from the corresponding author.

## Supplementary Materials

The following supporting information can be downloaded at https://www.mdpi.com/article/10.3390/ani15111629/s1. Table S1. Description of the characteristics and satellite image of each of the 12 estuaries sampled in southwestern Australia. Table S2. Details of the sampling regime used in each of the 12 estuaries and the mean and range of values recorded for a suite of water physicochemical parameters during sampling. Table S3. Percentage contribution of the ten most abundant taxa recorded in each salinity category. Table S4. The percentage frequency of occurrence of the ten most common taxa recorded in each salinity category. Figure S1. Raincloud plots, i.e., the combination of a scatter plot, probability density, and a box plot of the (a) salinity (ppt), (b) water temperature (°C), and (c) dissolved oxygen concentration (mgL^−1^) in each of the 12 estuaries.
