# Supplementary material for: Hypersalinity Drives Dramatic Shifts in the Invertebrate Fauna of Estuaries
Source: Animals (Basel). 2025 Jun 1;15(11):1629. doi: 10.3390/ani15111629 (PMC12153658; doi:10.3390/ani15111629)
Supplement: Supplementary file 1 [file animals-15-01629-s001.zip › animals-3636772-supplementary.pdf]

Roots *et al.* (2025). Hypersalinity drives dramatic shifts in the invertebrate fauna of estuaries

Supplementary materials

Table S1. Description of the characteristics and satellite image of each of the 12 estuaries sampled in southwestern Australia. Data taken from [35,36] and the images from Google Earth.

Swan-Canning Estuary
Location and surface area: 32.05°S, 115.73°E; 38.1 km^2.
Bar type: Permanently-open.
Freshwater source: Swan-Avon and Canning rivers with a mean annual flow of 600,000 ML. Median annual rainfall in the catchment of 409 mm.
Depth: Most < 5 m, with deeper areas in the entrance channel reaching ~20 m and lower parts of the main basin.
Substrate type: Coarse-fine sands in the shallow waters of the entrance channel and basins, with silt in the deeper waters. Silt, mud and gravel in the upper reaches.
Vegetation: *Halophila ovalis* is the dominant seagrass throughout the basins of the estuary, but other species such as *Zostera* spp., *Ruppia megacarpa* and *Halophila decipens* can be found in the entrance channel.

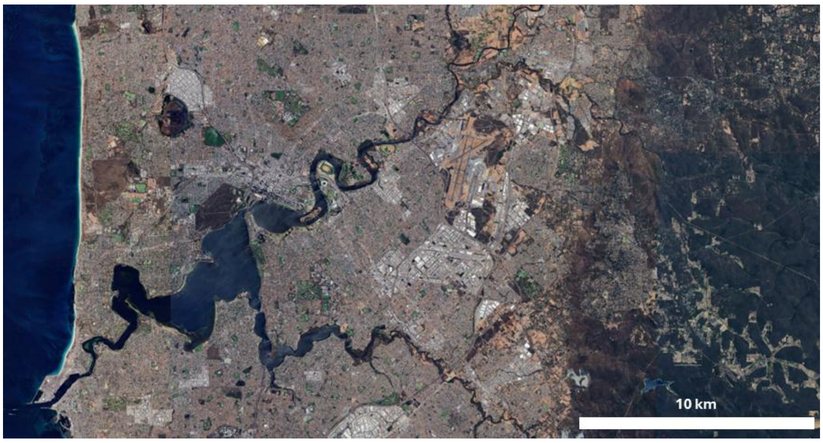

Peel-Harvey Estuary
Location and surface area: 32.61° S, 115.64° E; 133.6 km^2.
Bar type: Permanently-open. One natural mouth in Peel Inlet and an artificial entrance channel (Dawesville Cut) at the north end of the Harvey Estuary.
Freshwater source: Serpentine, Murray and Harvey rivers with a mean annual flow of 810,000 ML. Median annual rainfall in the catchment of 662 mm.
Depth: Mostly < 2 m, with extensive shallow marginal shoals.
Substrate type: Coarse-fine sands in the shallow waters of the entrance channel and basins, with silt in the deeper waters.
Vegetation: A suite of seagrasses, mainly dominated by *Ruppia* spp., with *Halophila*, *Heterozostera* and *Posidonia australis.* Significant accumulations of the green alga *Willeella brachyclados* (formerly *Cladophora montagneana*) can occur.

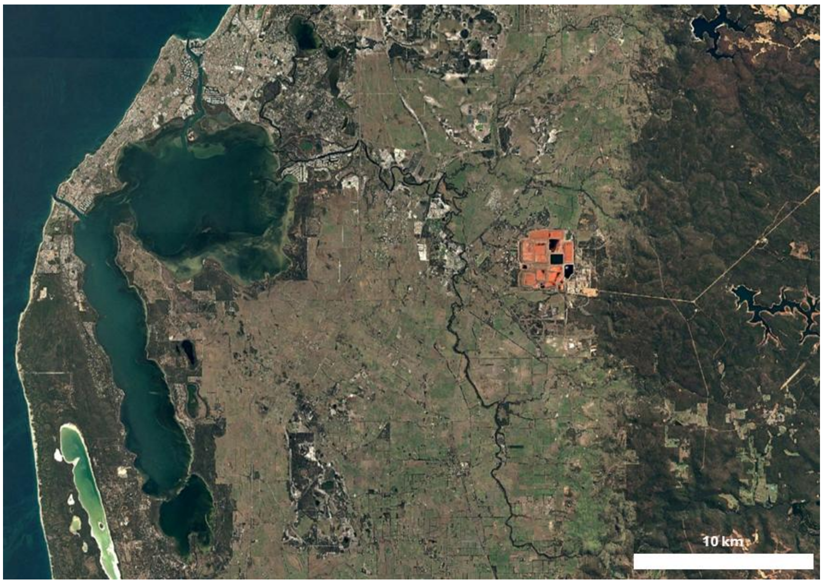

Vasse-Wonnerup Estuary

Location and surface area: 33.63° S, 115.40° E; 6.1 km².

Bar type: Annually-open. Bar breaches naturally, typically in winter/spring, but can be breached artificially (usually in summer) to help maintain good water quality.

Freshwater source: Vasse, Sabina, Abba and Ludlow rivers, with a mean annual flow of 40,300 ML. Median annual rainfall in the catchment of 905 mm.

Depth: Maximum of 2 m, with parts of the upper reaches drying out in summer/autumn.

Substrate type: Mainly coarse-fine sands, with localised areas of black suldific ooze on the upstream side of the Vasse tidal exclusion barrier.

Vegetation: *Ruppia megacarpa* and *Ruppia polycarpa* are found throughout the system. *Stuckenia pectinata* and *Althenia cyulindrocarpa* are found in the lower and upper reaches, respectively. Blooms of the macroalgae *Ulva* spp. and *Cladophora* spp. can occur.

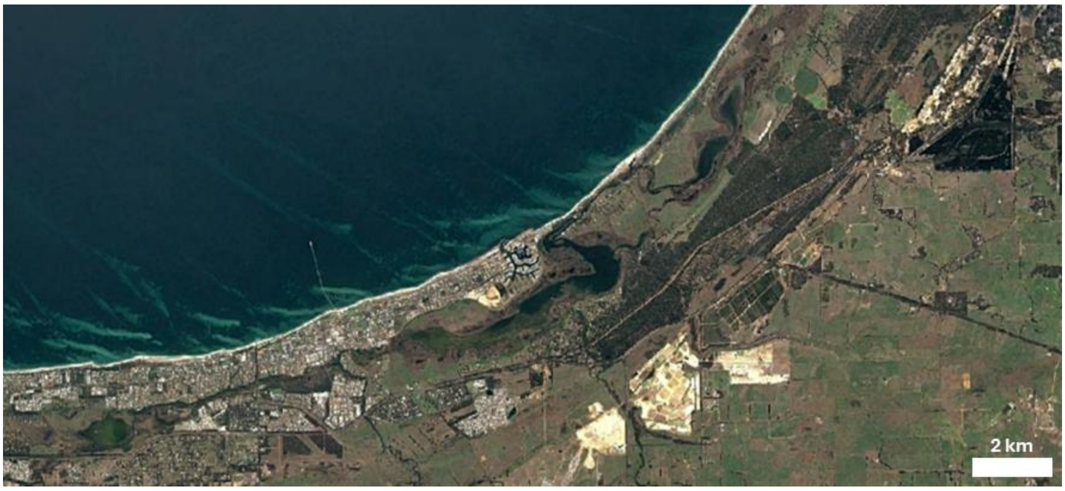

Broke Inlet

Location and surface area: 34.92° S, 116.44° E; 45.6 km².

Bar type: Annually-open. The bar extends 2 m above sea level and breaches naturally, typically in winter/spring.

Freshwater source: Shannon, Forth and Inlet rivers, with a mean annual flow of 162,000 ML. Median annual rainfall in the catchment of 1,331 mm.

Depth: Extensive marginal shoals (< 2m) in the basin, with a maximum depth of ~5-6 m in the entrance channel.

Substrate type: Medium to coarse siliceous sand throughout the marginal shoals, with sand and silt in deeper waters of the entrance channel and basins, respectively.

Vegetation: Mainly *Ruppia megacarpa. Lamprothanium papulosum* is present in some areas.

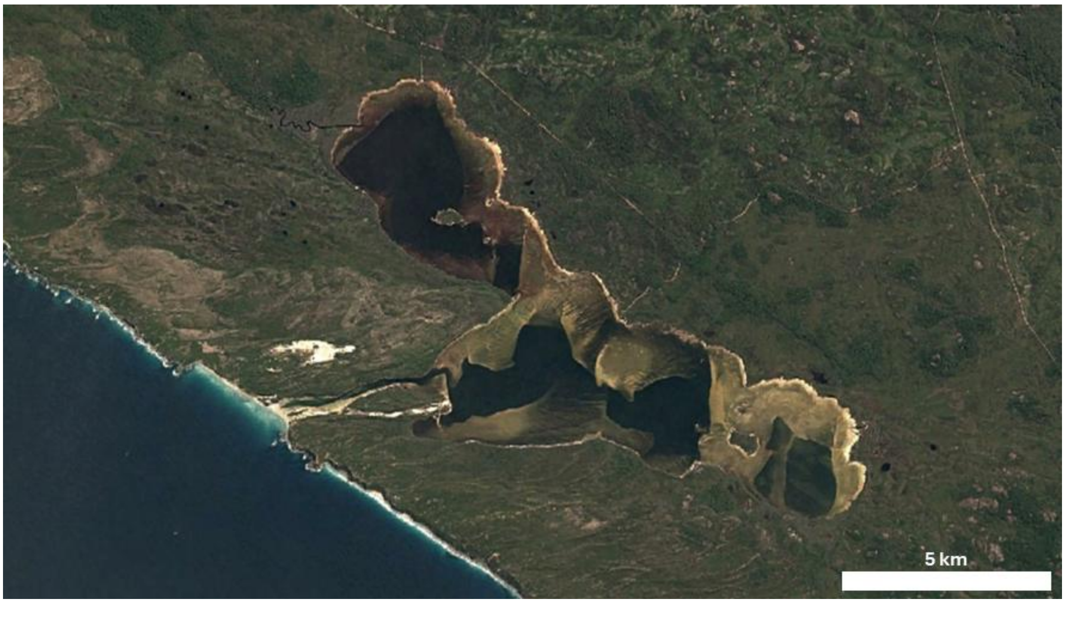

Torbay Inlet

Location and surface area: 35.03° S, 117.67° E; 0.9 km^2.
Bar type: Annually open. Bar (up to 2 m high) breaches several times a year, either naturally or artificially, to prevent flooding of adjacent land.
Freshwater source: Torbay Drain and Marbelup Brook, with a mean annual flow of 75,300 ML. Median annual rainfall in the catchment of 943 mm.
Depth: Up to 2 - 3 m.
Substrate type: Mainly sand.
Vegetation: The red alga *Gracilaria* spp. has been recorded previously, but not during sampling.

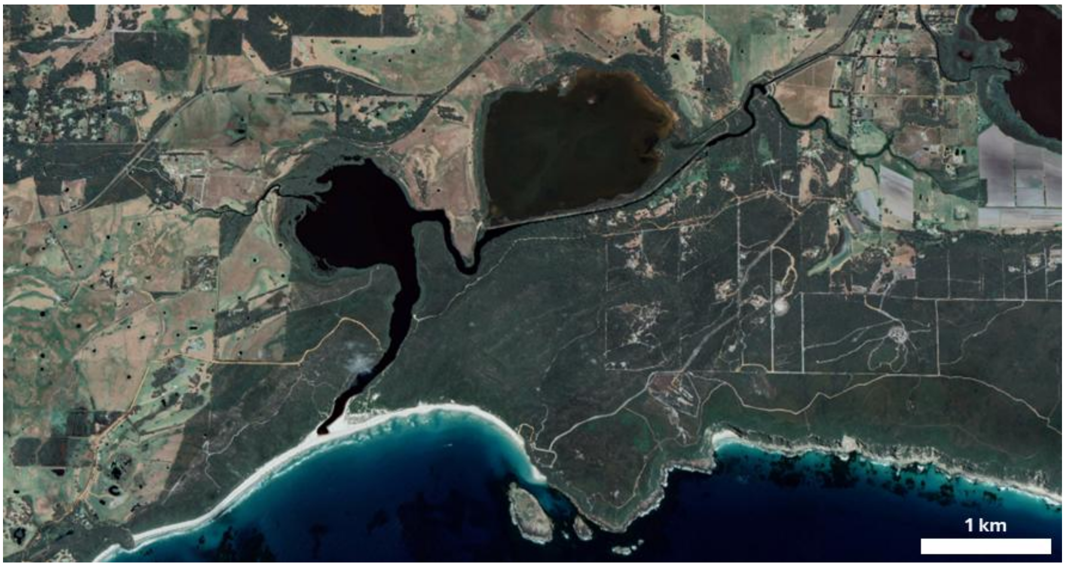

Oyster Harbor

Location and surface area: 34.99° S, 117.94° E; 17.7 km^2.
Bar type: Permanently-open.
Freshwater source: Kalgan and King rivers, with a mean annual flow of 97,200 ML. Median annual rainfall in the catchment of 949 mm.
Depth: Average of ~ 5 m but reaches 10 m in some areas.
Substrate type: Medium-coarse to fine sands, silty sands with broken marine shells. Emergent granite boulders support oysters, and beds of cockles can be present in the lower reaches.
Vegetation: *Posidonia australis* is typically in shallow waters and *Posidonia sinuosa* in deeper waters. The macroalgae *Cladophora prolifera* can occur.

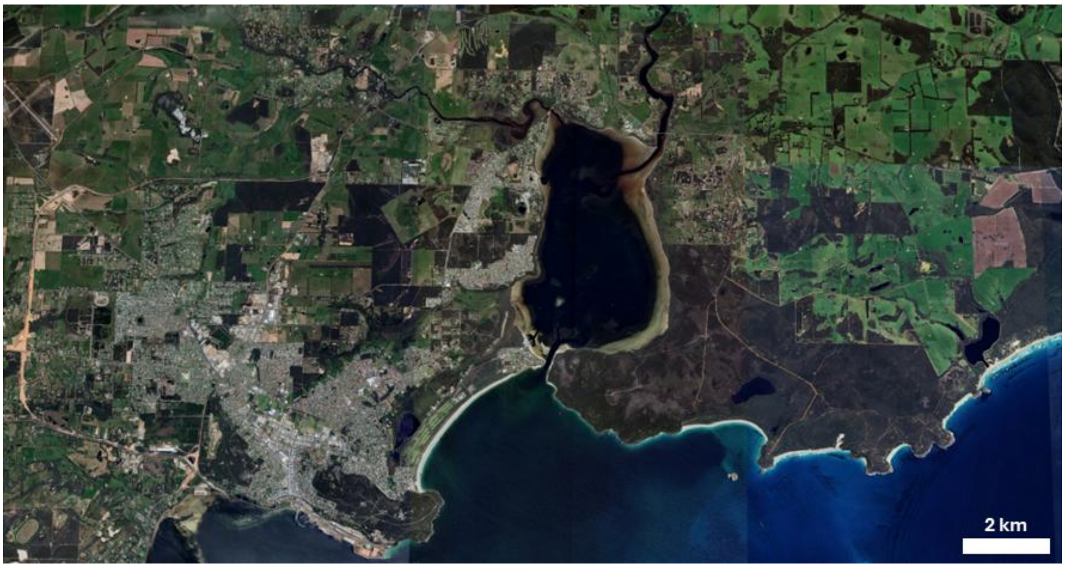

Taylor Inlet
Location and surface area: 34.99° S, 118.06° E; 0.5 km².
Bar type: Annually-open.
Freshwater source: Three small creeks. Mean annual flow of 1,400 ML and a median annual rainfall in the catchment of 800 mm.
Depth: Most ~ 2 m deep, but reaches 5 m in places.
Substrate type: Mainly sand.
Vegetation: Dense stands of *Ruppia* sp. and charophytes were recorded during sampling.

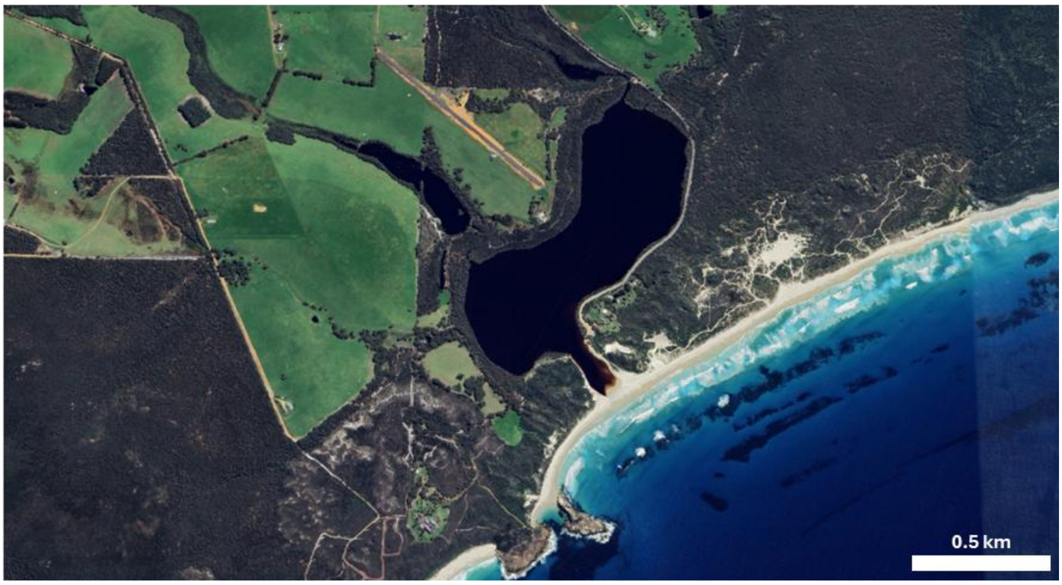

Normans Inlet
Location and surface area: 34.55° S, 118.13° E; 0.2 km².
Bar type: Annually-open. Bar can reach 1.5 m high.
Freshwater source: One river, with a mean annual flow of 1,800 ML. Median annual rainfall in the catchment is 810 mm.
Depth: ~ 1 m deep, but decreases after bar breaching.
Substrate type: Firm sandy-muddy sediments in the basin and muddy further upstream.
Vegetation: Dense seagrass beds of *Ruppia* spp. in the basin and *L. papulosum* in the upstream reaches.

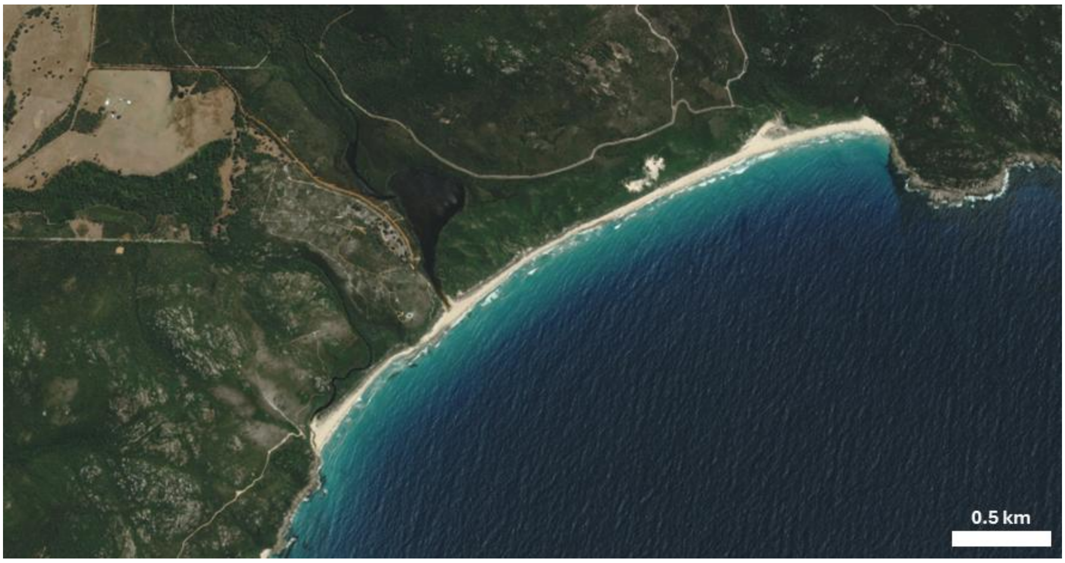

Waychinicup Estuary

Location and surface area: 34.54° S, 118.19° E; 0.1 km^2.
Bar type: Permanently-open as the estuary lies in a gorge carved between two large granite headlands.
Freshwater source: Waychinicup River, with a mean annual flow of 1,700 ML. Median annual rainfall in the catchment is 760 mm.
Depth: Reaches a maximum of ~ 22 m are the entrances and decreased upstream to 2 – 3 m.
Substrate type: Coarse sand and gravel in the lower and basin regions, and muddy sediment further upstream.
Vegetation: Shorelines are dominated by Phaeophyceae and Rhodophyta macroalgae, with expansive beds of *Posidonia* spp. and *Halophila* spp. seagrasses.

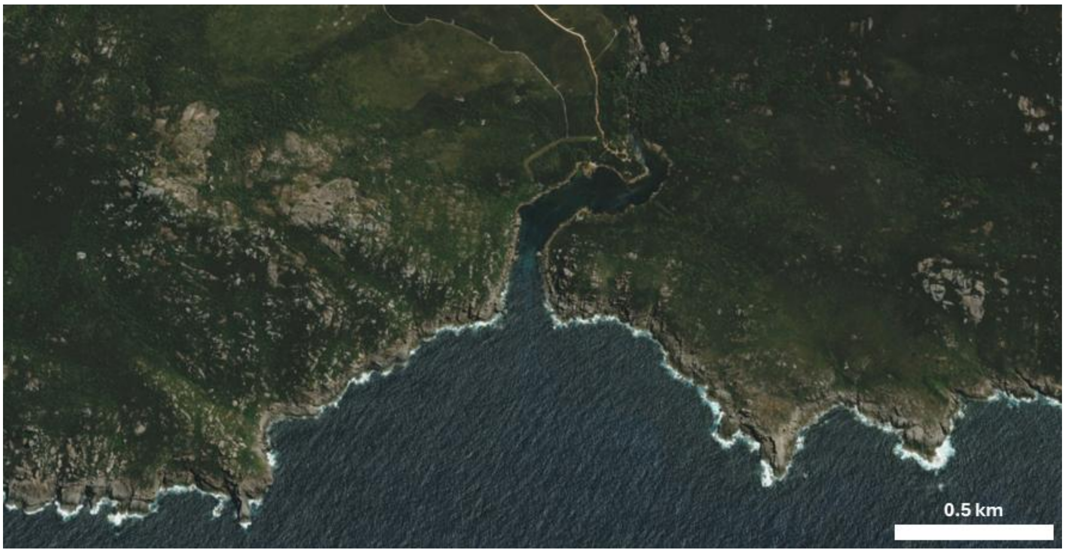

Cordinup River

Location and surface area: 34.42° S, 118.34° E; 0.1 km^2.
Bar type: Bar breaches every 2-3 year, but can be annually-opening.
Freshwater source: Cordinup River, with a mean annual flow of 1,700 ML. Median annual rainfall in the catchment is 610 mm.
Depth: ~2 m deep during bar closure, but ~0.5 m when bar is open.
Substrate type: Predominantly sandy bottomed, with muddy soil in riverine reaches.
Vegetation: *Ruppia* sp. and *Lamprothanium* spp. Are present throughout the basin and upper regions.

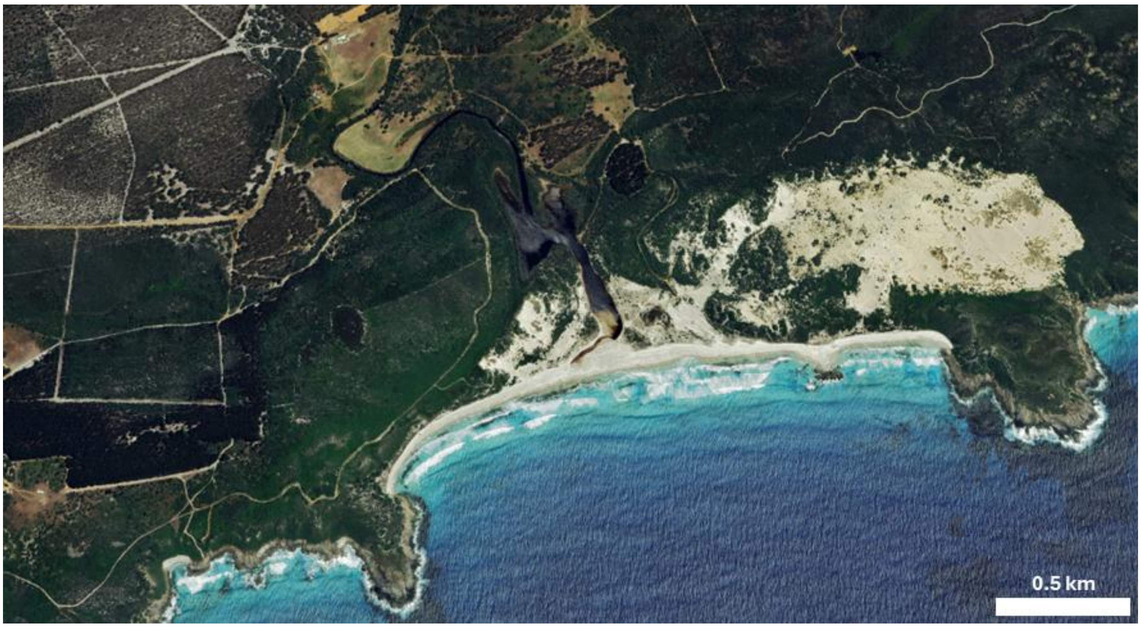

Cheyne Inlet
Location and surface area: 34.36° S, 118.46° E; 0.2 km^2.
Bar type: Annually open, with a 1.5 m tall bar.
Freshwater source: Eyre River, with a mean annual flow of 1,800 ML. Median annual rainfall in the catchment is 610 mm.
Depth: <1m deep.
Substrate type: The lagoon has silty sand whereas the riverine has both mud and sand. The bar has fine white and quartz sand.
Vegetation: Vegetation such as *Ruppia* spp., used to be common upstream from the bar. *Cladophora* spp., and *Enteromorpha paradoxa* can be abundant in the lagoon.

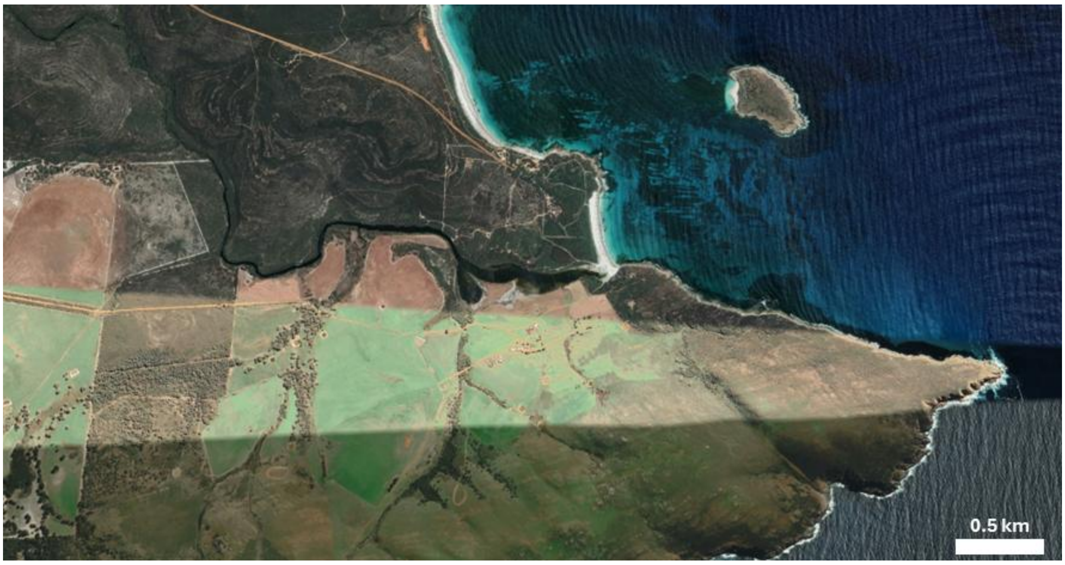

Beaufort Inlet
Location and surface area: 34.45° S, 118.88° E; 6.5 km^2
Bar type: Normally closed with a large bar that has a height up to 3.5 m.
Freshwater source: Pallinup River, with a mean annual flow of 36,000 ML. Median annual rainfall in the catchment is 410 mm. River is saline.
Depth: 1-2 m deep basin, with deeper reaches >7m.
Substrate type: The bar comprises of coarse quartz sand and shell material. Upper region is slightly gravelly sand whereas the rest is sand.
Vegetation: No macrophytes recorded.

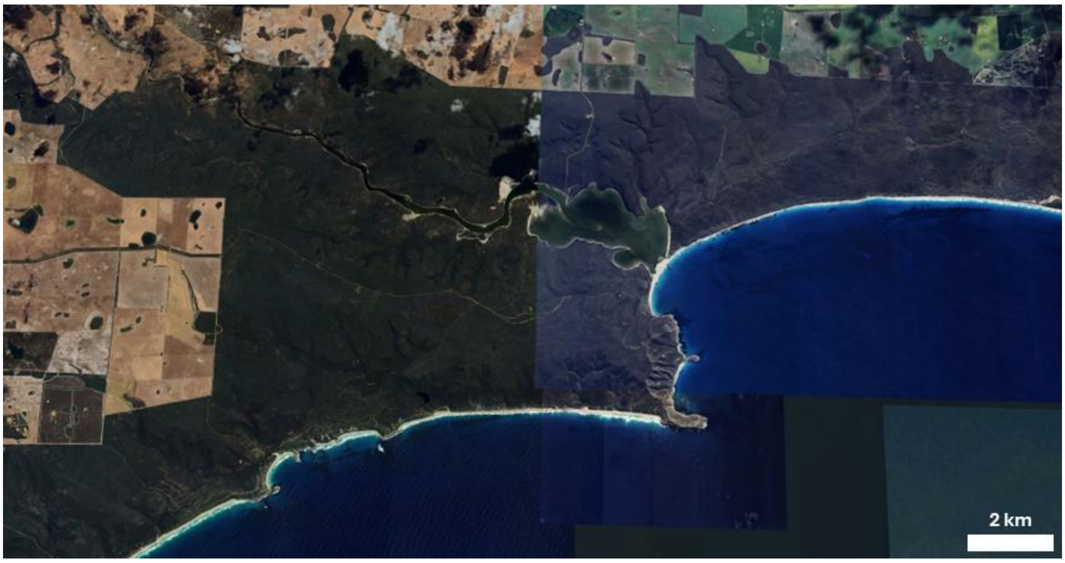

Table S2. Details of the sampling regime used in each of the 12 estuaries and the mean and range of values recorded for a suite of water physicochemical parameters during sampling. *n* = number of samples. Modified from [36].

	Region	Sites	Timing	*n*	Method	Salinity (ppt)		Temperature (°C)		DO (mgL^{-1})		Reference
						Mean	Range	Mean	Range	Mean	Range	
Swan-Canning Estuary	Upper	5	11 months (2010/11)	309	Ekman grab (225 cm^2)	19.0	4.0-32.5	21.2	11.6-29.7	3.3	0.2-11.0	[37]
	Lower	4	4 seasons (2023/24)	80	Corer (96 cm^2)	34.5	19.1-39.5	22.4	14.3-30.9	8.3	6.4-10.5	[38]
	Lower	6	1 season (2023)	90	Corer (96 cm^2)	35.2	34.8-35.6	25.1	23.5-27.6	8.0	6.1-10.6	[39]
Peel-Harvey Estuary	All	60	2 seasons (2017/18)	120	Corer (28 cm^2)	26.7	2.1-56.2	21.3	13.9-28.9	5.8	0.1-10.6	[40]
Vasse-Wonnerup Estuary	All	30	15 seasons (2017-20)	344	Ekman grab (225 cm^2)	26.5	0.6-96.5	20.5	12.0-34.3	8.8	0.6-18.2	[41-42]
Broke Inlet	All	40	5 seasons (2007/08)	480	Corer (96 cm^2)	22.9	2.1-45.1	18.1	12.6-28.3	6.0	2.5-20.4	[43]
Torbay Inlet	All	12	4 seasons (2020)	48	Corer (96 cm^2)	14.1	1.0-34.7	19.1	12.4-25.7	8.0	2.2-11.6	[44]
Oyster Harbour	All	12	4 seasons (2020)	48	Corer (96 cm^2)	34.1	17.4-37.6	17.6	12.3-22.3	8.3	1.5-12.2	[44]
Taylor Inlet	All	12	4 seasons (2020)	48	Corer (96 cm^2)	20.0	16.1-24.2	17.5	12.5-22.5	9.6	6.1-16.3	[44]
Normans Inlet	All	12	4 seasons (2020)	48	Corer (96 cm^2)	4.2	2.4-6.2	16.9	12.5-22.0	8.4	4.3-10.4	[44]
Waychinicup Estuary	All	12	4 seasons (2020)	48	Corer (96 cm^2)	31.9	3.4-35.4	18.3	15.5-21.5	8.1	6.3-10.3	[44]
Cordinup River	All	12	4 seasons (2020)	48	Corer (96 cm^2)	12.6	7.9-19.3	17.6	14.3-23.2	8.4	4.4-13.9	[44]
Cheyne Inlet	All	12	4 seasons (2020)	48	Corer (96 cm^2)	42.5	33.0-54.3	18.0	13.4-25.7	7.6	3.7-10.7	[44]
Beaufort Inlet	All	12	11 times (2020-23)	132	Corer (96 cm^2)	56.3	3.2-121.8	17.7	8.6-25.5	7.4	3.6-16.2	[4]

Table S3. Percentage contribution of the ten most abundant taxa recorded in each salinity category (10 ppt intervals; i.e. 0 = 0.0 to 9.9, 10 = 10 to 19.9 ppt, and so on).

**0**	
Capitella spp.	16.73
Arthritica semen	10.18
Corophium minor	6.93
Chironominae spp.	5.97
Simplisetia aequisetis	4.68
Potamopyrgus spp.	4.26
Cyathura hakea	3.87
Mytilocypris mytiloides	3.30
Ascorhis occidua	3.27
Desdemona ornata	3.16

**10**	
Capitella spp.	19.26
Pseudopolydora cf. *kempi*	13.03
Simplisetia aequisetis	11.48
Arthritica semen	8.62
Prionospio cirrifera	5.62
Cyathura hakea	4.89
Desdemona ornata	4.38
Scoloplos normalis	4.21
Chironominae spp.	4.18
Fluviolanatus subtortus	3.32

**20**	
Prionospio cirrifera	15.54
Capitella spp.	14.45
Arthritica semen	10.93
Pseudopolydora cf. *kempi*	7.22
Simplisetia aequisetis	7.03
Desdemona ornata	4.70
Corophium minor	4.04
Potamopyrgus spp.	3.91
Armandia intermedia	3.55
Scoloplos normalis	2.87

**30**	
Capitella spp.	21.16
Simplisetia aequisetis	12.00
Arthritica semen	8.96
Corophium minor	6.31
Armandia intermedia	4.28
Cyathura hakea	4.15
Scoloplos normalis	3.02
Prionospio cirrifera	2.84
Hiatula biradiata	2.50
Heteromastus filiformis	2.27

**40**	
Capitella spp.	46.52
Corophium minor	9.57
Arthritica semen	5.40
Simplisetia aequisetis	3.80
Potamopyrgus spp.	3.63
Prionospio cirrifera	3.30
Armandia intermedia	2.79
Scoloplos normalis	2.69
Barnardomelita matilda	1.73
Cyathura hakea	1.60

**50**	
Capitella spp.	37.82
Tanytarsus barbitarsis	31.96
Potamopyrgus spp.	7.86
Procladius sp.	5.49
Simplisetia aequisetis	2.75
Tanea sp.	2.08
Scoloplos normalis	1.49
Arthritica semen	1.37
Oligochaeta spp.	1.29
Prionospio cirrifera	1.25

**60**	
Tanytarsus barbitarsis	45.67
Capitella spp.	30.59
Procladius sp.	9.51
Potamopyrgus spp.	7.88
Mytilocypris ambiguosa	2.65
Diptera spp.	1.82
Mytilocypris mytiloides	0.76
Leptoceridae spp.	0.57
Simplisetia aequisetis	0.19
Trichoptera spp.	0.15

**70**	
Capitella spp.	42.70
Leptoceridae spp.	33.15
Potamopyrgus spp.	15.84
Chironominae spp.	6.77
Alboa worooa	1.54

**80**	
Tanytarsus barbitarsis	47.37
Capitella spp.	27.56
Potamopyrgus spp.	17.53
Oligochaeta spp.	3.37
Diptera spp.	1.88
Berosus spp.	0.67
Leptoceridae spp.	0.67
Culicidae spp.	0.48
Procladius sp.	0.48

**90**	
Tanytarsus barbitarsis	78.87
Capitella spp.	10.86
Ceratopogonidae spp.	5.51
Corophium minor	3.03
Diptera spp.	1.31
Leptoceridae spp.	0.24
Berosus spp.	0.12
Potamopyrgus spp.	0.06

**100**	
Tanytarsus barbitarsis	87.33
Ceratopogonidae spp.	5.89
Capitella spp.	5.10
Pseudopolydora cf. *kempi*	1.25
Diptera spp.	0.38
Grandidierella spp.	0.05

**110**	
Tanytarsus barbitarsis	100.00

**≥120**	
Tanytarsus barbitarsis	100.00

Table S4. Percentage frequency of occurrence the ten most common taxa recorded in each salinity category (10 ppt intervals; i.e. 0 = 0.0 to 9.9, 10 = 10 to 19.9 ppt, and so on).

**0**	
Capitella spp.	45.2
Arthritica semen	40.6
Simplisetia aequisetis	33.9
Scoloplos normalis	28.7
Chironominae spp.	22.3
Corophium minor	21.4
Paracorophium excavatum	20.9
Pseudopolydora cf. *kempi*	18.8
Fluviolanatus subtortus	18.0
Desdemona ornata	17.1

**10**	
Capitella spp.	53.8
Simplisetia aequisetis	45.4
Arthritica semen	38.3
Scoloplos normalis	34.6
Pseudopolydora cf. *kempi*	34.1
Desdemona ornata	24.1
Prionospio cirrifera	19.9
Fluviolanatus subtortus	17.3
Cyathura hakea	17.1
Chironominae spp.	16.3

**20**	
Capitella spp.	46.0
Arthritica semen	46.0
Prionospio cirrifera	41.0
Simplisetia aequisetis	35.4
Pseudopolydora cf. *kempi*	29.1
Scoloplos normalis	27.2
Desdemona ornata	23.8
Fluviolanatus subtortus	18.5
Corophium minor	18.3
Grandidierella spp.	16.9

**30**	
Capitella spp.	64.1
Simplisetia aequisetis	56.9
Scoloplos normalis	41.5
Arthritica semen	36.2
Corophium minor	33.5
Hiatula biradiata	23.9
Pseudopolydora cf. *kempi*	23.8
Grandidierella spp.	23.2
Oligochaeta spp.	22.1
Prionospio cirrifera	21.4

**40**	
Capitella spp.	74.2
Corophium minor	38.2
Simplisetia aequisetis	32.6
Scoloplos normalis	27.0
Arthritica semen	24.7
Potamopyrgus spp.	18.0
Prionospio cirrifera	16.9
Armandia intermedia	15.7
Barnardomelita matilda	13.5
Procladius sp.	13.5

**50**	
Capitella spp.	55.9
Tanytarsus barbitarsis	32.4
Scoloplos normalis	26.5
Simplisetia aequisetis	20.6
Arthritica semen	14.7
Potamopyrgus spp.	14.7
Oligochaeta spp.	8.8
Diptera spp.	8.8
Chironominae spp.	8.8
Procladius sp.	8.8

**60**	
Tanytarsus barbitarsis	47.8
Capitella spp.	43.5
Potamopyrgus spp.	30.4
Procladius sp.	21.7
Mytilocypris ambiguosa	13.0
Mytilocypris mytiloides	8.7
Diptera spp.	8.7
Chironominae spp.	8.7
Simplisetia aequisetis	4.3
Leptoceridae spp.	4.3

**70**	
Capitella spp.	100.0
Potamopyrgus spp.	80.0
Leptoceridae spp.	60.0
Chironominae spp.	40.0
Alboa worooa	20.0

**80**	
Capitella spp.	75.0
Potamopyrgus spp.	50.0
Tanytarsus barbitarsis	50.0
Oligochaeta spp.	25.0
Culicidae spp.	25.0
Diptera spp.	25.0
Berosus spp.	25.0
Leptoceridae spp.	25.0
Procladius sp.	25.0

**90**	
Tanytarsus barbitarsis	90.9
Capitella spp.	54.5
Diptera spp.	36.4
Ceratopogonidae spp.	18.2
Corophium minor	9.1
Berosus spp.	9.1
Leptoceridae spp.	9.1
Potamopyrgus spp.	9.1

**100**	
Tanytarsus barbitarsis	75.0
Ceratopogonidae spp.	12.5
Capitella spp.	8.3
Pseudopolydora kempi	4.2
Grandidierella spp.	4.2
Diptera spp.	4.2

**110**	
Tanytarsus barbitarsis	90.0

**≥120**	
Tanytarsus barbitarsis	100.00

Figure S1. Raincloud plots, i.e. the combination of a scatter plot, probability density and a box plot of the (a) salinity (ppt), (b) water temperature (°C) and (c) dissolved oxygen concentration (mgL^{-1}) in each of the 12 estuaries. Data for each estuary is shown in a different colour; SC, Swan-Canning Estuary; PH, Peel-Harvey Estuary; VW, Vasse-Wonnerup Estuary; Br, Broke Inlet; Tb, Torbay Inlet; OH, Oyster Harbour; Tl, Taylor Inlet; No, Normans Inlet; Wy, Waychinicup Estuary; Co, Cordinup River; Cy, Cheyne Inlet; Bf, Beaufort Inlet. Taken from [36].

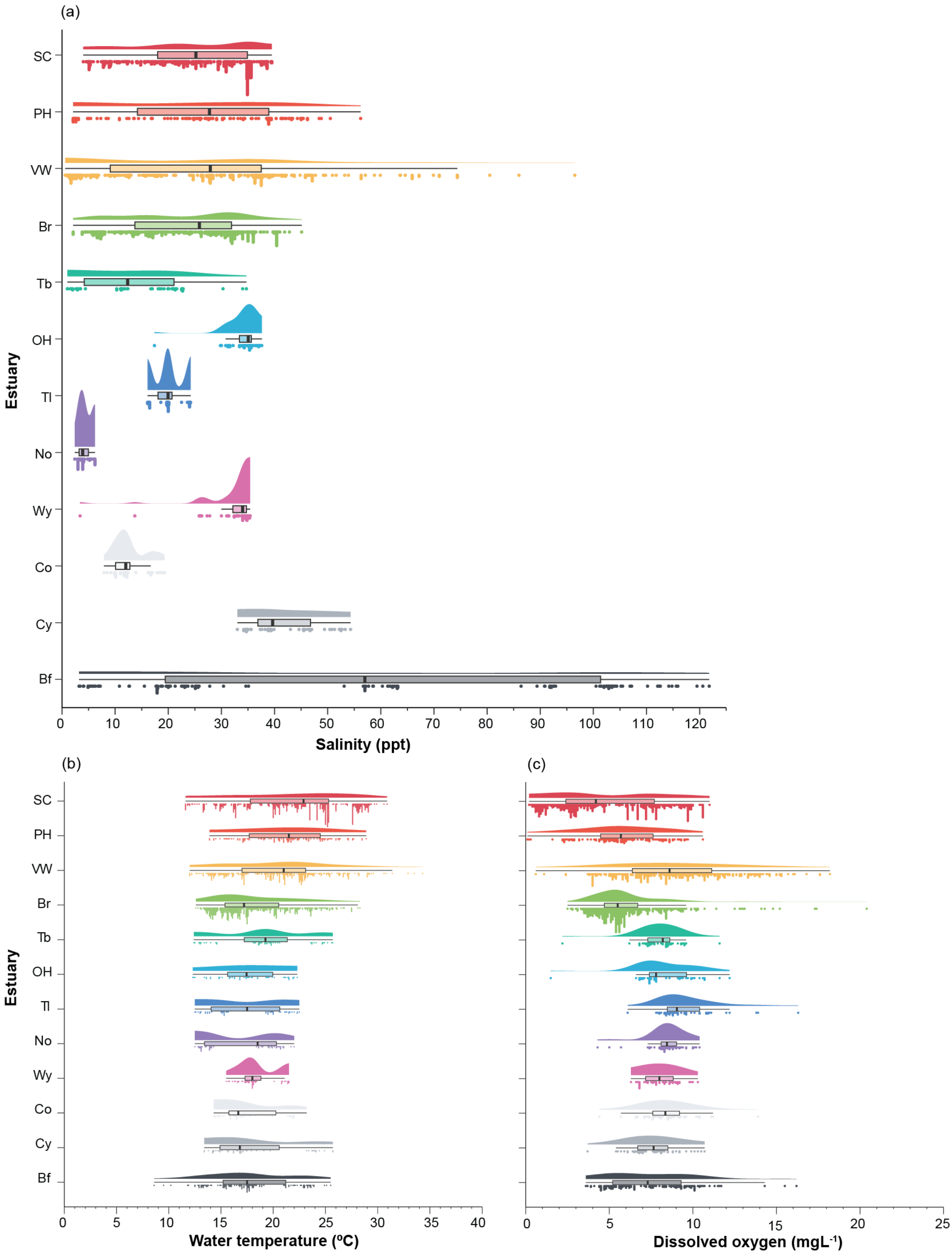

References

4. Cronin-O'Reilly, S.; Krispyn, K.N.; Maus, C.; Standish, R.J.; Loneragan, N.R.; Tweedley, J.R. Empirical evidence of alternative stable states in an estuary. Science of The Total Environment **2024**, 954, 176356,
35. Brearley, A. *Ernest Hodgkin's Swanland*, 1st ed.; University of Western Australia Press: Crawley, **2005**; p. 550.
36. Lim, R.; Fourie, S.A.; Stout, E.J.; Roots, B.J.; Cronin-O'Reilly, S.; Rodgers, E.M.; Tweedley, J.R. Testing the Remane diagram: occurrences of benthic macroinvertebrates in oligohaline to hyperhaline salinities. *Water* **2025**, *17*, 1642.
37. Tweedley, J.R.; Hallett, C.S.; Warwick, R.M.; Clarke, K.R.; Potter, I.C. The hypoxia that developed in a microtidal estuary following an extreme storm produced dramatic changes in the benthos. *Marine and Freshwater Research* **2016**, *67*, 327-341.
38. Lim, R. Multidecadal Changes in the Benthic Macroinvertebrates Assemblages of the Swan-Canning Estuary. Murdoch University, Perth, Australia, **2025**.
39. Stout, E. Benthic macroinvertebrate communities of the Swan Estuary Marine Park. Murdoch University, Perth, Australia, **2025**.
40. Cronin-O'Reilly, S. Benthic community structure, health and function of a microtidal estuary in south-western Australia. Murdoch, Perth, Western Australia, **2021**.
41. Tweedley, J.R.; Cronin-O'Reilly, S.; Cottingham, A.; Beatty, S.J. *Vasse-Wonnerup Integrated Monitoring Review of 2017-20: Benthic macroinvertebrate component*; Murdoch University, Perth, Western Australia: Report for the Department of Water and Environmental Regulation, **2021**; p. 65.
42. Cronin-O'Reilly, S.; Cottingham, A.; Kalnejais, L.H.; Lynch, K.; Tweedley, J.R. Tidal exclusion barriers fragment an invertebrate community into taxonomically and functionally distinct estuarine and wetland assemblages. *J. Mar. Sci. Eng.* **2025**, *13*, 635.
43. Tweedley, J.R. The relationships between habitat types and faunal community structure in Broke Inlet, Western Australia. PhD, Murdoch University, Australia, Perth, **2011**.
44. Fourie, S.A. Benthic macroinvertebrate faunas of microtidal estuaries in Albany, south-western Australia. Murdoch University, Perth, Australia, **2024**.
